# Visual Stimulation Under 4 Hz, Not at 10 Hz, Generates the Highest-Amplitude Frequency-Tagged Responses of the Human Brain: Understanding the Effect of Stimulation Frequency

**DOI:** 10.1523/ENEURO.0426-24.2025

**Published:** 2025-06-12

**Authors:** Talia L. Retter, Christine Schiltz

**Affiliations:** ^1^Department of Behavioral and Cognitive Sciences, Institute of Cognitive Science & Assessment, University of Luxembourg, Esch-sur-Alzette L-4366, Luxembourg; ^2^Université de Lorraine, CNRS, IMoPA, Nancy F-54000, France

**Keywords:** Fourier spectrum analysis, harmonic frequencies, rhythmic visual stimulus, steady-state, stimulation rate, visual evoked potentials

## Abstract

In frequency tagging, visual stimulation at a frequency (F) of ∼10 Hz has long been known to generate the highest-amplitude response at* F *in the frequency domain over the human occipital cortex with electroencephalogram and other high temporal-resolution methods. Brain responses are indeed commonly assessed simply at* F* (i.e., the first harmonic = 1*F*), under the assumption that the response is represented at a single frequency, i.e., “steady-state” or approximately sinusoidal in terms of amplitude over time. This condition is met at stimulus presentation frequencies above ∼4–8 Hz in the visual modality; consequently, frequency tagging has often been limited to F above this “floor.” Here, we support a less-common perspective, that frequency-tagged responses do not need to be steady-state, such that slower F are valid. In this case, it has been shown that is not appropriate to measure nonsinusoidal responses at only *F* but that nonsinusoidal responses can still be analyzed simply and advantageously in the frequency domain through baseline-corrected amplitude summation across harmonics (*F* *+* 2*F* *+* 3*F*… = *F*_s_). Critically, we demonstrate that although the highest-amplitude *F* response occurs at F = 10 Hz, the highest-amplitude *F*_s_ response occurs at approximately F < 4 Hz. We use this example toward understanding the effect of stimulation frequency on response amplitude and discuss its caveats and limitations. We address what defines an “optimal” stimulation frequency (note: it may not always be the F yielding the highest-amplitude response) and reflect on considerations when choosing a stimulation frequency in different contexts.

## Significance Statement

Over nearly a century of research, it has generally been thought that steady-state brain responses should be targeted in frequency-tagging, through a floor stimulation frequency (F) of ∼4–8 stimuli/second (Hz). In a different conceptual framework, we propose that there is no need to pursue steady-state brain responses. At F's < 4 Hz, response amplitude is more distributed across multiple harmonics (1*F*, 2*F*, 3*F*, etc.), but it has been demonstrated that overall response amplitude can be measured through baseline-corrected harmonic amplitude summation (*F*_s_). While the highest-amplitude occipital responses of the human brain occur at 1*F* = 10 Hz, the highest-amplitude comprehensive responses occur at *F_s_* < 4 Hz. This implies that slower F may be considered optimal in some contexts of fundamental and applied research.

## Introduction

Frequency tagging is an experimental approach in which stimuli that are presented across time periodically, at a chosen stimulation frequency, F, elicit periodic brain responses that can be analyzed advantageously in the frequency domain at *F* (i.e., the first harmonic = 1*F; *please note the notation italics) and its higher harmonics, 2*F*, *3F*, etc. Frequency tagging has been applied with multiple modalities (visual, auditory, somatosensory…) and recording methods [electroencephalogram (EEG), electroretinogram, functional magnetic resonance imaging…], in various populations (human adults, infants, nonhuman primates…; Regan, 1989; Norcia et al., 2015; Retter et al., 2021b). The fundamental goal of frequency-tagging research is to develop understanding of brain function through responses to exogenous stimulation; frequency tagging also has practical applications in clinical assessment and brain–computer/machine interfaces (BCI/BMI).

The frequency-domain analysis of frequency-tagged responses confers important advantages of the technique: (1) frequency-tagged responses can be identified and measured objectively, exactly at the fundamental stimulation frequency, *F*, and its higher harmonics, 2*F*, 3*F*, etc., in the frequency domain; (2) responses to multiple stimuli may be continuously “tagged” with different stimulation frequencies simultaneously within a testing sequence, leading to independent signals or potentially interactions at intermodulation terms; (3) frequency-domain responses present with a high signal-to-noise ratio (SNR), since the frequency-tagged response is localized in specific frequency bins, while “noise” activity is broadband; (4) stimulation is traditionally fast, contributing to a high information transfer rate and short testing duration; and (5) due to the simplicity of stimulation and analysis with modern equipment, elicited responses can be measured with high automaticity, requiring minimal system configuration and user training (Regan, 1972; 1989; Wang et al., 2008; Rossion, 2014; Norcia et al., 2015). In light of these advantages, usage of the frequency-tagging technique should be more widespread.

One barrier to implementing frequency tagging may be the outstanding question of which stimulation frequency to use and concerns that the stimulation frequency will affect the recorded brain responses. Choosing a stimulation frequency is fundamental to the frequency tagging technique (e.g., “One of the key questions in SSVEP recording is the choice of the stimulus frequency”: Norcia et al., 2015, p. 4; “The stimulus frequency is one of the most important factors in SSVEP BCI applications”: Reitelbach and Oyibo, 2024, p. 16). In general, there is confusion about which stimulation frequency should be selected: “…there is no consensus regarding the optimal frequencies…”: Kuś et al., 2013, p. 2; “…the choice of stimulation frequencies remains somewhat arbitrary”: Köster et al., 2023, p. 101315). In one striking example to the contrary, there is a conception that visual stimulation at ∼10 Hz is optimal, as it yields the highest-amplitude EEG responses at *F* over the occipital cortex.

The pervasive notion of 10 Hz as the optimal stimulation frequency will be examined in the following section (Stimulation Frequency Can Affect Brain Responses: Is Visual Stimulation at 10 Hz Optimal?), beginning with its origins before turning to its conceptual assumptions (regarding “steady-state” responses) and related methodological foundation (i.e., analysis simply at *F*). In subsequent unravelling of this notion, we will take the less-common perspective that frequency-tagged responses do not need to be steady-state and that non-steady-state responses can be analyzed with baseline-corrected amplitude summation across harmonics (*F* *+* 2*F* *+* 3*F*… = *F*_s_; Retter et al., 2021b). We will demonstrate that while the highest-amplitude responses at *F* occur at F = 10 Hz over the occipital cortex, the highest-amplitude responses overall, at *F*_s_, occur at F < 4 Hz. In the next section (Visual Stimulation Under 4 Hz, Not at 10 Hz, Generates the Highest-Amplitude Responses), we will show that high *F*_s_ amplitude is stable across F under 4 Hz and is not an artifact of baseline activity/noise. We will subsequently pivot to discuss understanding the effect of stimulation frequency on response amplitude through the relation with response temporal dynamics, which determine the response frequency content (Understanding the Effect of Stimulation Frequency on Response Amplitude through the Relation with Response Temporal Dynamics). We will address what defines an “optimal” stimulation frequency and reflect on considerations when choosing a stimulation frequency in different contexts (Considerations When Choosing a Stimulation Frequency). Finally, we will discuss caveats (Caveats) and summarize our conclusions (Conclusion). Overall, the goal is to understand the impact of stimulation frequency on the amplitude of brain responses.

## Stimulation Frequency Can Affect Brain Responses: Is Visual Stimulation at 10 Hz Optimal?

**Figure 1.**
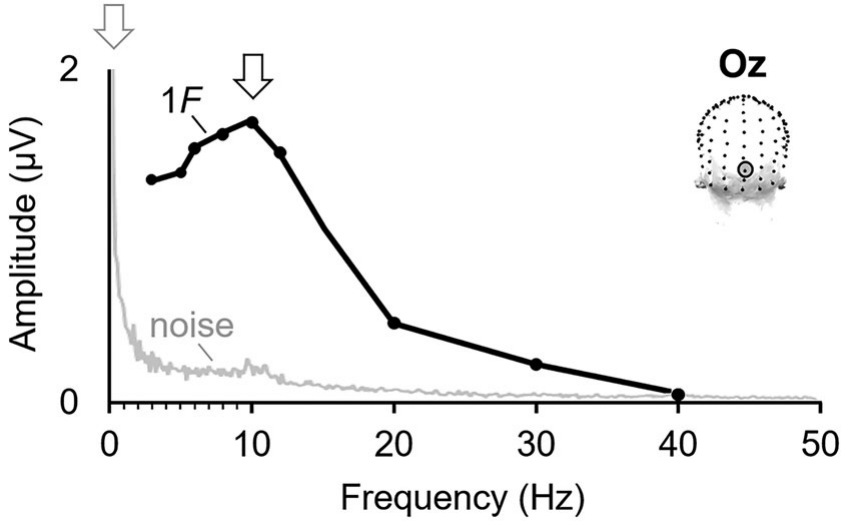
EEG responses at *F* as a function of visual stimulation frequency. Data derived from Retter et al. (2021a) (electrode Oz; *N* = 16 people; stimuli = natural face images; Butterworth 4th-order filter from 0.05–140 Hz). The amplitude of only the first harmonic (*F*, i.e., 1*F*) is plotted (black line), as in many analyses in the literature. The maximal *F* response occurs to visual stimulation at 10 Hz here (arrow). Note that the amplitude pattern of responses to stimulation does not follow the trend of baseline noise activity (light gray line; from the 40 Hz stimulation condition), which is highest close to 0 Hz and falls steeply until near 0.5–1 Hz, and then tends to decrease gradually, often with a smaller local peak around the alpha band (8–12 Hz) range.

During the earliest human EEG recordings, a prominent, spontaneous electrical potential at ∼10 Hz was discovered with recordings over the occipital cortex (Berger, 1929). It was also discovered that “regular potential waves at frequencies other than 10 a second can be induced by flicker”: periodic visual stimulation at frequency F, tested between 7 to 25 Hz, elicited periodic brain responses at *F* (Adrian and Matthews, 1934, p. 377). Further, it was observed that the frequency of stimulation had an effect on the elicited response: “The flicker rhythms are shown most clearly with rates between 10 and 20 a second (fig. 19)” (Adrian and Matthews, 1934, p. 380). The result of a clear, i.e., high amplitude, brain response to visual stimuli over the occipital cortex at ∼10 Hz was replicated in later studies using frequency-domain analyses: “…a stimulus frequency which lies within a range centered near 10 Hz evokes a response of large amplitude compared with that at neighbouring frequencies” (as tested and reviewed in Regan, 1972, p. 77; Fig. 1; see also Kamp et al., 1960; van der Tweel, 1964; Regan, 1966a). (It may be noted that the pattern of response amplitude to stimulation as a function of frequency deviates from the pattern of baseline “noise” (baseline brain activity and noise): indeed, the peak visual response ∼10 Hz is commonly reported in terms of baseline-corrected data, such as SNR and baseline-subtracted amplitude.)

Even today, there is a conception that the visual stimulation frequency eliciting the highest-amplitude response, often taken as the “optimal” stimulation frequency, is ∼10 Hz (sometimes extending higher, up to ∼15–18 Hz) for frequency-tagged responses of the human brain, as recorded occipitally with high temporal resolution techniques such as EEG/MEG (e.g., “…the optimal range of frequencies, which is 12–18 Hz…”: Kuś et al., 2013, p. e77536; “…frequencies from 10 to 16 Hz resulted in the best SSVEP response…”: Reitelbach and Oyibo, 2024, p. 10; see also Herrmann, 2001; Pastor et al., 2003; Heine and Meigen, 2004; Wang et al., 2006; Vialatte et al., 2009; Bakardjian et al., 2010; Zhu et al., 2010; Herbst et al., 2013; and ∼40 Hz for auditory stimuli: Galambos et al., 1981; Ross et al., 2000; Pastor et al., 2002). Although a wide range of stimulation frequencies have been tested, often between ∼3–20 Hz, and most often above 8–10 Hz (Norcia et al., 2015; 1–15 Hz in developmental studies: Fig. 4 of Köster et al., 2023), in a recent review, ∼90% of studies were reported to use at least one stimulation frequency between 10–12 Hz (Reitelbach and Oyibo, 2024).

However, the determination of an optimal visual stimulation frequency of 10 Hz may be incorrect, since it is based on three misconceptions: (1) an unnecessary definition of frequency-tagged brain responses as “steady-state” or cyclical, i.e., more-or-less sinusoidal; (2) the related assessment of responses at only the first harmonic frequency, *F* (i.e., without considering higher harmonics: 2*F*, 3*F*, etc.); and (3) confounding the stimulation and response frequencies. These related misconceptions will be addressed in turn in the following three subsections completing this section.

### Misconception 1: steady-state responses should be targeted with F above 4 Hz

In principle there is no minimal stimulation frequency (F) imposed by frequency-tagging as long as the stimulation is periodic, above 0 Hz. However, there is a “minimal theoretical limit (4 Hz)” to the stimulation frequency in the popular understanding that frequency-tagged responses should be “steady-state” (Lopez-Gordo et al., 2011, p. 129; Birca et al., 2010; Çetin et al., 2020). The term “steady-state” defines elicited responses to periodic stimulation that are constant in terms of mean amplitude and phase over time, i.e., with a repetitive wave pattern, and is the basis for the terms steady-state visual-evoked potentials/responses (SSVEP/R; Regan, 1966a; 1977; 1989; Heinrich, 2010; Norcia et al., 2015) and auditory steady state (evoked) potentials/responses (ASS(E)P/R; Geisler, 1960; Watkin, 2008). Such terms (also “traveling wave responses”: Engel et al., 1997) are commonly used for the frequency-tagging approach in general, although they limit frequency-tagged responses to this specific condition. Steady-state responses may be even further limited to the condition that their amplitude over time is modulated by a dominant fundamental sinusoidal component: a sinusoidal shape is often taken as the epitome of the steady-state response (Müller et al., 1997; Heinrich, 2010; Norcia et al., 2015).

Going well beyond terminology, there is a longstanding popular assumption that all frequency-tagged responses must be steady-state. It is even sometimes claimed that steady-state responses are required for frequency-domain analysis (Çetin et al., 2020). Steady-state frequency-tagged responses have been taken as fundamentally different from acyclical frequency-tagged responses, without a clear rationale: originally, these responses were considered to be the most continual in the time domain and so most precisely represented in the frequency domain (Regan, 1972, 1989); the dominant ongoing justification appears to be the benefit that steady-state responses may be analyzed simply in the frequency domain, through analysis only at *F* (but this is at a cost: addressed below).

The implication of considering that all frequency-tagged responses should be steady-state is that there is a stimulation frequency floor, of ∼4–8 Hz (sometimes set at 4 Hz, and sometimes higher, up to ∼8 Hz) in the visual modality. Indeed, steady-state frequency-tagged responses are commonly defined as existing only above an F floor. For several examples: (1) “Steady state evoked responses are elicited by stimulating the eyes with a light flickering faster than approximately 5 Hz” (Wilson and O’Donnell, 1986, p. 57); (2) “SSVEP in response to a visual stimulus that is repeated at steady rate of 8–10 Hz or more…recorded from the scalp as a nearly sinusoidal oscillatory response…” (Morgan et al., 1996, p. 4770); (3) “SSVEP represents oscillatory electrical potential that is elicited in the brain when the subject is visually watching a stimulus that is flickering at a frequency of 6 Hz or above” (Xu et al., 2023, p. 1); and (4) as noted by Vialatte et al. (2009, p. 400), “…many recent publications still convey the notion that SSVEP can only be recorded above 3 or 4 Hz” and Norcia et al., “while SSVEPs can be recorded at a wide range of frequencies, in most studies the stimulus frequency (i.e., presentation rate) tends to be above 8–10 Hz…” (2015, p. 2).

**Figure 2.**
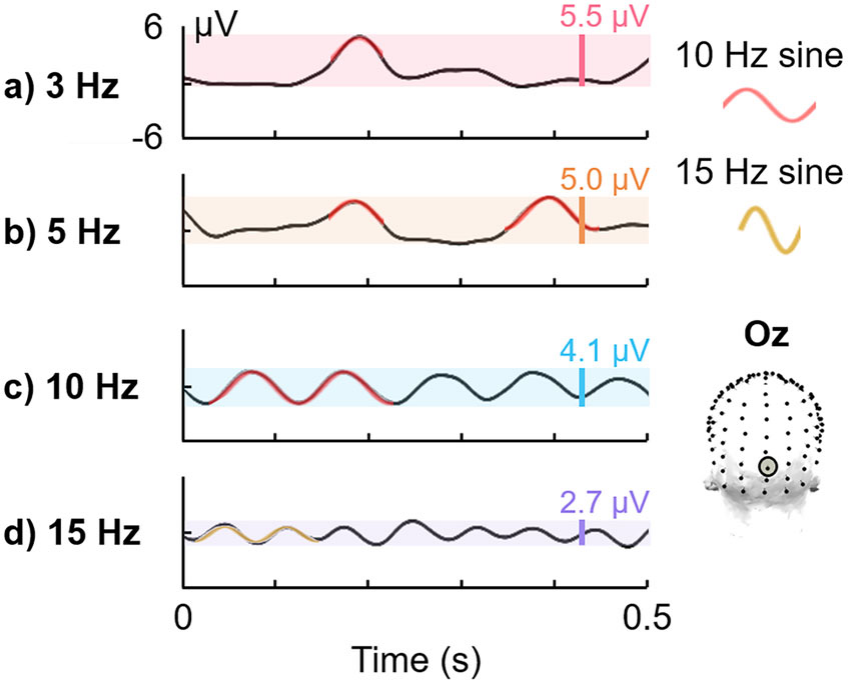
EEG responses to periodic visual stimulation in the time domain. Data derived from Retter et al. (2021a) (electrode Oz; *N* = 16 people; stimuli = natural face images). The response amplitude range, decreasing as stimulation frequency increases, is highlighted in color and given in each panel. ***a***, ***b***, Some peak response deflections to stimulation at 3 and 5 Hz are fit approximately with segments of a 10 Hz sine wave (red highlights). ***c***, At 10 Hz stimulation, the response becomes cyclical and is fit well with a 10 Hz sine wave (2 cycles highlighted). ***d***, At 15 Hz stimulation, the cyclical response is condensed to 15 Hz (2 15 Hz cycles highlighted in yellow).

The reason that a minimal F is imposed for generating steady-state brain responses is that at such high stimulation frequencies, brain responses that may have shown more complex, i.e., acyclical, amplitude deflections over time at lower stimulation frequencies overlap in time enough to become cyclical, even nearly sinusoidal (Regan, 1989; see also Sokol, 1976; Zhou et al., 2016; for illustrations of the transition to approximately sinusoidal, from nonsinusoidal, responses as stimulation rate increases, see Fig. 2; also shown in Fig. 2 of van der Tweel, 1964; Fig. 9 of Sokol, 1976; Fig. 7 and Fig. 2 of Alonso-Prieto et al., 2013). As responses become less complex and more sinusoidal, the number of harmonic frequency responses decreases: again, if the response was entirely sinusoidal, there would only be amplitude at the *F* response frequency (further illustrations of increasing sinusoidal responses, including frequency-domain representations across harmonics: Fig. 6 of Zhou et al., 2016; Fig. 4 of Retter et al., 2021b). While there is no single definition of “high” F, these frequencies are evidenced by the emergence of more-or-less sinusoidal responses; this may occur at variable rates for different brain responses (Keysers and Perrett, 2002; Retter et al., 2020; see also Heinrich, 2010; Retter et al., 2021b).

Taking this into account, not only do we propose that frequency tagging does not need to be steady-state (implying that stimulation frequencies below 4 Hz are valid) but that presinusoidal stimulation frequencies (leading to multiharmonic frequency-domain responses; e.g., below ∼8–10 Hz for occipitally recorded responses to visual stimulation) may confer benefits for the quality of responses recorded. In the words of D. Regan: “…at high repetition rates only a few harmonic components fall within the brain's passband so that the steady-state EP [evoked potential] waveform is simpler than at low repetition rates… the simplicity of the response should, however, be regarded with caution because it is achieved by, in effect, filtering out information” (Regan, 1989, p. 35).

We take the position that there is no inherent necessity for frequency-tagged brain responses to be steady-state and that steady-state brain responses represent a subset of frequency-tagged responses without a distinctive functional basis or need for conceptual dissociation from acyclical responses generated at lower stimulation frequencies (see Norcia et al., 2015 and section 4.6 of Retter and Rossion, 2016). Note that this perspective is in line with the understanding of frequency-tagged responses as corresponding generally with superimposed, interfering event-related potential (ERP) responses, rather than having a distinct (oscillatory) functional basis (Bohórquez and Ozdamar, 2008; Capilla et al., 2011; Gaume et al., 2019; but addressing different perspectives: e.g., Makeig et al., 2002; Heinrich, 2010; Keitel et al., 2014; Heinrich et al., 2015).

Congruent with the perspective that steady-state responses are not required, there are also many terms for the frequency tagging approach that are agnostic to the shape of the brain responses elicited in terms of their amplitude over time. Some such terms reference the periodicity of the analyses, such as Fourier/frequency (domain) analysis/synthesis (Movshon et al., 1978; McKeefry et al., 1996; Bach and Meigen, 1999; Zhou et al., 2016) and frequency tagging (Tononi et al., 1998; Srinivasan et al., 1999), while others emphasize the periodicity of the stimulation, such as rhythmic sensory stimulation (Walter and Walter, 1949), (intermittent/repetitive) photic stimulation [(I/R)PS; Takahashi, 1999], and repetitive/rhythmic visual stimulus (RVS; Zhu et al., 2010; Köster et al., 2023; Reitelbach and Oyibo, 2024), or the speed of the stimulation, such as fast periodic visual stimulation (FPVS; Rossion, 2014). The multitude of terms for the frequency-tagging approach may appear arbitrary and redundant, but we suggest that the generic use of terms such as SSVEP has reinforced an unnecessary conception of all frequency-tagged responses as steady state or cyclical and terms that do not share this bias may be preferable (Rossion, 2014). Again, the implication is that when steady-state responses are not specifically targeted, lower F, below 4 Hz in the visual modality, are valid.

### Misconception 2: responses should be assessed only at the first harmonic frequency, *F*

When targeting approximately sinusoidal, steady-state brain responses through F above 4–8 Hz at minimum, brain responses may be represented in the frequency domain in few harmonic components, even predominately at the first harmonic frequency, *F*, since a sinusoid is represented in the frequency domain at a single frequency (Zhou et al., 2016; Retter et al., 2021b). This is seen as an important advantage of the steady-state approach in particular, making response analysis targeting only *F* simple and straightforward (Regan, 1972; 1989; Livingstone et al., 1991; Fig. 2 of Alonso-Prieto et al., 2013; Fig. 6 of Zhou et al., 2016; Fig. 4 of Retter et al., 2021b).

In contrast, stimulating at lower frequencies elicits frequency-tagged responses that are often acyclical and represented across multiple harmonic frequencies, e.g., *F*, 2*F*, 3*F*, etc. (Walter et al., 1946; Brazier, 1964; van der Tweel and Lunel, 1965; Regan, 1989; Fawcett et al., 2004; Vialatte et al., 2009; Heinrich et al., 2015; Retter and Rossion, 2016; Zhou et al., 2016; Retter et al., 2021b). The presence of multiharmonic responses to F below ∼4 Hz may make the response analysis appear more complicated (Regan, 1966a; 1989; Bach and Meigen, 1999; Heinrich, 2009; Norcia et al., 2015; Retter and Rossion, 2016; Zhou et al., 2016). In the case of harmonic responses with amplitudes similar or greater than that of the fundamental, “measurements of the synchronous [*F*] component alone could therefore give a misleading impression of the amplitude of the total evoked response*…*” (Regan, 1966a, p. 246). Indeed, when multiharmonic responses are evident, an analysis on only the first harmonic is incomplete and does not correspond to amplitude ranges apparent in the time domain, despite a pervasive application of studies’ analyses limited to *F*, even for multiharmonic responses (Retter et al., 2021b).

One longstanding limitation in analyzing multiharmonic responses was that a method for addressing them has not been applied systematically across the literature (Retter et al., 2021b). It has even been thought that “there is no simple rule that would tell us how to combine the amplitude values at different harmonics into one single number that could be used as a measure of neural activity” (Heinrich, 2010, p. 209). Indeed, harmonic responses have commonly been left out of the response analysis, and when they have been included, the methodology has been highly variable: higher harmonics have often been reported individually or combined through different approaches, e.g., root-mean-square summation or averaging in terms of amplitude, power, or SNR (reviewed in Retter et al., 2021b). Still, including higher harmonic responses is valuable: this has been shown to be important for measuring brain responses (Cebulla et al., 2006; Heinrich, 2009; Heinrich et al., 2009; Tlumak et al., 2011; Gaume et al., 2019; Retter and Rossion, 2016; Stothart et al., 2017; Netto et al., 2023; review: Retter et al., 2021b), as well as for benefiting clinical diagnosticity (Falsini et al., 1999; Lazarev et al., 2001; Van der Donck et al., 2019; Vettori et al., 2019; David et al., 2025), and improving brain–computer interface signal detection and classification (Davila et al., 1998; Muller-Putz et al., 2005; Chen et al., 2015; Çetin et al., 2020).

We are in agreement with a simple analysis for combining multiharmonic response amplitudes into a single measure: baseline-corrected harmonic amplitude summation (Retter et al., 2021b). The summation of harmonic response amplitude has not only been applied in previous studies (Janz et al., 2001; Cheng et al., 2002; Retter and Rossion, 2016; summed power: Wang et al., 2008; Zhang et al., 2011), but it has also been advocated for in relation to other approaches (Heinrich, 2009; Rossion et al., 2020; Retter et al., 2021b). In this approach, a baseline correction in the frequency domain, such as a simple subtraction of an estimated noise level (e.g., the average of a range of surrounding noise bins), is applied to each harmonic. The number of harmonics to include in the analysis remains a variable to consider (Retter et al., 2021b), but it is not a critical one: in the absence of signal at the frequencies of interest, a baseline subtraction ideally sets the amplitude to zero, largely compensating for the irregularity of noise amplitude across the frequency spectrum. The resulting baseline-corrected harmonic amplitudes are then summed together to form a single, comprehensive response amplitude measurement, *F*_s_.

Due to the availability and modern ease of multiharmonic response analysis, visual stimulation above ∼4–8 Hz, to target steady-state brain responses, no longer provides a methodological advantage. Indeed, the fundamental advantages of frequency tagging are present for non-steady-state frequency-tagged responses as well. Further, slower stimulation frequencies provide an additional advantage, in that more complex brain responses can be characterized through multiharmonic and more nuanced time-domain response analyses.

### Misconception 3: the optimal (i.e., highest-amplitude) *F* response frequency equals the optimal stimulation frequency

**Figure 3.**
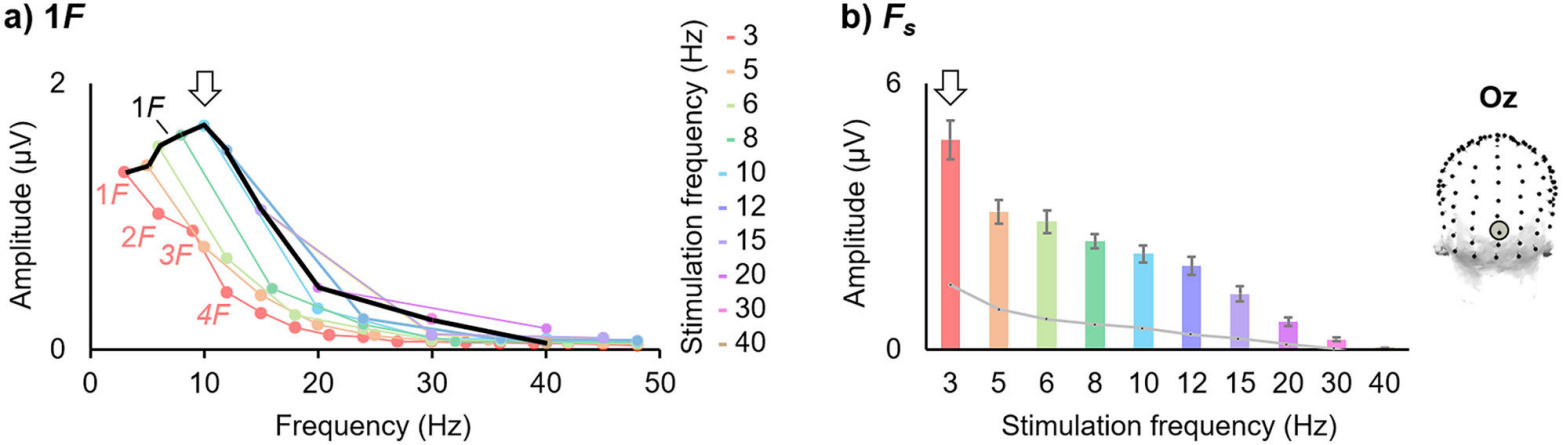
EEG responses as a function of visual stimulation frequency (Oz). Data derived from Retter et al. (2021a) (electrode Oz; *N* = 16 people; stimuli = natural face images). ***a***, As in Figure 1, the amplitude of only the first harmonic (1*F*, i.e., *F*) is plotted in black, leading to an apparent signal peak ∼10 Hz over the occipital cortex (marked here with an arrow). Additionally, the missing information of the higher harmonic frequency response amplitudes within each stimulation frequency condition is included here in colored lines (see the key); the first four harmonics are labeled illustratively for the 3 Hz stimulation frequency (1*F* = 3 Hz; 2*F* = 6 Hz; 3*F* = 9 Hz; 4*F* = 12 Hz). ***b***, In a recommended analysis, the response amplitude is summed across harmonic frequencies (*F*_s_; *F*'s below 50 Hz for each stimulation condition; see the colored line plots in panel ***a***), leading to the largest response amplitude at the lowest stimulation frequency tested, 3 Hz (marked with an arrow; error bars indicate ±1 SEM across participants; noise level (with which to correct the signal measurement) given by a light gray line).

In line with the earliest human EEG studies mentioned previously, it is generally the case that the highest amplitude or SNR of the brain response over the occipital cortex occurs at ∼10 Hz (up to ∼15 Hz) to visual stimuli; however, importantly, this refers to an analysis on only the first harmonic frequency, *F* (i.e., 1*F*; Regan, 1966a; 1989; Gao et al., 2003; Pastor et al., 2003; Fig. 5 of Ding et al., 2006; Wang et al., 2006; Pastor et al., 2007; Vialatte et al., 2009; Gulbinaite et al., 2019; reviews: Zhu et al., 2010; Reitelbach and Oyibo, 2024). Again, it is important to observe that the pervasive analysis at only a single harmonic, *F*, in line with the conception of frequency-tagged brain responses as approximately sinusoidal, may be largely appropriate at and above 10 Hz, but it is likely not appropriate for lower stimulation frequencies, when highly distributed, multiharmonic responses are elicited (Retter et al., 2021b). An analysis at only *F* misses important information about the response amplitude represented at higher harmonics, particularly for lower stimulation frequencies (Fig. 3*a*).

Critically, while the highest response amplitude at *F* may occur at 10 Hz over the occipital cortex, this does not imply that the optimal stimulation frequency is 10 Hz. When frequency-tagged responses of the human brain are analyzed across multiple harmonics (*F*_s_), the largest amplitude does not occur to visual stimulation at 10 Hz, but rather below ∼4 Hz (Fig. 3*b*; see also Fig. 2 of Lazarev et al., 2001; Alonso-Prieto et al., 2013; Retter and Rossion, 2016; Fig. 4 of Gaume et al., 2019; Retter et al., 2020; 2021a; Fig. 3 of Gu et al., 2024; with auditory stimuli: Tlumak et al., 2011).

Empirically, following baseline-corrected harmonic amplitude summation, the largest *F*_s_ response amplitude to visual stimulation is evident clearly at the lowest stimulation rate tested under ∼4 Hz, with the amplitude falling quickly above that range (3 Hz: Fig. S3A of Retter et al., 2020; 3 Hz: Fig. 4D of Retter et al., 2021a; 4 Hz: Fig. 5 of Marchive et al., 2024; 0.75 Hz with auditory stimulation: Fig. 4 of Tlumak et al., 2011; 1 Hz in a 1*F* analysis for text stimuli: Yeatman and Norcia, 2016). Studies in which the slowest visual stimulation rates tested were between 5–9 Hz, when addressing multiple harmonics in the frequency-domain and/or investigating response amplitudes in the time domain, have also reported the largest responses at the slowest frequency tested, rather than in a 10–15 Hz window (Müller et al., 1997; Fawcett et al., 2004; Kaspar et al., 2010; see Fig. 7 of Lin et al., 2012; Gruss et al., 2012; Nakanishi et al., 2014; Chai et al., 2017; Wang et al., 2021; *F* analysis: Livingstone et al., 1991; Shyu et al., 2013; Zheng et al., 2021; better time-domain object decoding at F = 5 than 20 Hz: Grootswagers et al., 2019). Indeed, the relative amplitude ranges of time-domain responses are more consistent with the amplitude of *F*_s_ than *F* response amplitude quantifications (compare Fig. 2 with Fig. 3: the time-domain range of response amplitude decreases as stimulation frequency increases from 3–15 Hz, as is the case for *F*_s_ but not *F* response amplitude measurements; Retter et al., 2021b).

It has been proposed that responses in different frequency ranges may reflect somewhat different brain processes and/or source locations (Regan, 1972; Sokol, 1976; Pastor et al., 2003). Perhaps in this vein, approximate frequency ranges of functionally distinctive processing subsystems were defined in foundational work by D. Regan for responses, not stimulation, approximately encompassing low (7–10 Hz), middle (12–25 Hz), and high (30–60 Hz) ranges (Regan, 1972; Regan, 1989), roughly corresponding to endogenous alpha (8–12 Hz), beta (12–30 Hz), and gamma (30–100 Hz) oscillation frequency ranges (Berger, 1930; Jasper and Andrews, 1938). Again, the response frequency and the stimulation frequency should be not be confounded: while the *F* response frequency equals the stimulation frequency F, the higher harmonic responses, affected by their frequencies in turn, are also often a considerable part of the response. The stimulation frequency producing the highest-amplitude *F* response may therefore not be the stimulation frequency producing the highest-amplitude response overall (*F*_s_). In the case of visual stimulation measured over the occipital cortex, the highest-amplitude *F* EEG response occurs at approximately F = 10 Hz, but the highest-amplitude *F*_s_ response occurs at approximately F < 4 Hz.

## Visual Stimulation Under 4 Hz, Not at 10 Hz, Generates the Highest-Amplitude Responses

In the previous section, we proposed that the highest-amplitude responses of the human brain to visual stimulation occur under ∼4 Hz with frequency tagging (Figs. 2, 3). In the following, we take this proposition two steps further, in addressing that stable response amplitudes may be found under 4 Hz (see Visual stimulation under 4 Hz yields stable, high-amplitude *F_s_* responses) and that the highest-amplitude responses occurring under 4 Hz is not merely a consequence of the highest baseline activity and noise presenting at very low frequencies (see The highest-amplitude responses under 4 Hz are not accounted for by high baseline activity/noise).

### Visual stimulation under 4 Hz yields stable, high-amplitude *F_s_* responses

We have proposed that frequency-tagging does not need to be steady-state with visual stimulation rates above ∼4–8 Hz but that opposite to this, stimulation below this range produces the highest-amplitude brain responses overall (*F_s_*). At such low stimulation frequencies, temporally distanced brain responses are not suppressed by interference from successive-response overlap that ultimately yields cyclical responses, but may be recorded with their full, complex expression (Fig. 2; Bandettini et al., 1993; Kovács et al., 1995; Keysers et al., 2001; Keysers and Perrett, 2002; Retter and Rossion, 2016; Retter et al., 2020; Retter et al., 2021b). Under this logic, responses may occur with the highest amplitude, i.e., least suppression, below ∼3–4 Hz, because 250–333 ms is the duration in which the majority of the activity is contained for recordings of many visually elicited brain responses. For example, ERPs to many visual stimuli have been traditionally plotted with a minimum time axis limit of ∼350–400 ms: since responses typically onset with a latency of ∼50–100 ms, this leaves approximately a 250–350 ms window minimum for responses analysis (VanRullen and Thorpe, 2001; Johnson and Oshausen, 2003; Rossion and Jacques, 2008; see also Retter and Rossion, 2016).

Due to a lack of successive response interference below ∼4 Hz, the baseline-corrected response amplitude may occur fully and thus be stable, i.e., fairly independent of stimulation frequency. For example, if an uninterrupted response to a visual stimulus is recorded every 1.5 s or 2 s, the response (component) amplitudes in the time domain have little reason to vary, and so the response amplitude as quantified in the frequency domain should not vary (e.g., equivalent amplitudes to frequency-tagged responses at 0.7 Hz and nonperiodic stimulation occurring on average at 0.7 Hz: Quek and Rossion, 2017).

**Figure 4.**
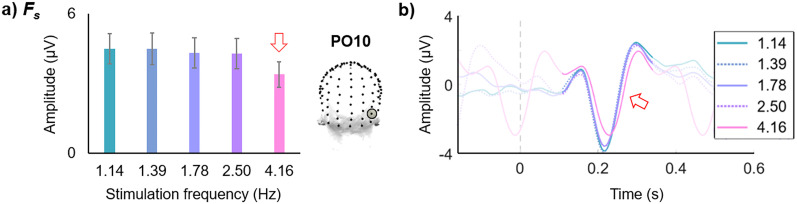
EEG responses as a function of low visual stimulation frequency. Data derived from Retter and Rossion (2016) (electrode PO10; *N* = 16 people; stimuli = natural face vs object images; *F_s_* summed up to 19 Hz; as in Fig. 3 of that study; and its Fig. 2 for frequency-domain harmonic distributions and Fig. 4 for time-domain amplitudes). ***a***, The baseline-subtracted amplitude of *F_s_* is stable across the lowest stimulation frequencies, from 1.14 to 2.50 Hz, and is decreased only at the higher stimulation frequency of 4.16 Hz (red arrow). ***b***, The time-domain deflections’ amplitude is stable across the lowest stimulation frequencies, from 1.14 to 2.50 Hz, and is decreased only at the highest stimulation frequency of 4.16 Hz (red arrow; full-contrast time period represents one full cycle at 4.16 Hz = 240 ms), in correspondence with the *F_s_* frequency-domain amplitudes.

Indeed, stable *F_s_* EEG amplitudes were present for four stimulation frequencies ranging from 1.14 to 2.50 Hz (Fig. 4*a*; Retter and Rossion, 2016). In contrast, when F was increased so that responses were temporally overlapping and interfering (F = 4.16 Hz), the *F_s_* response amplitude (Fig. 4*a*; and time-domain amplitude range: Fig. 4*b*) was decreased. For other examples: similar responses to deviant words were reported at F = 2 and 3 Hz in Wang et al., 2021; and stable *F_s_* EEG amplitudes were also reported to 0.75–1.25 Hz auditory stimulation (already decreasing at 2.5 Hz and above: Tlumak et al., 2011).

A couple exceptions to this response amplitude stability at low F's may be when the stimulation frequency is extremely low, below ∼0.5 Hz, since the baseline noise level may be extremely high and variable; and when very short recording times are used, in which case a higher information transfer rate at higher stimulation frequencies may confer a more considerable benefit in signal versus noise measurement. Still, we propose that the highest-amplitude responses to visual stimulation occur under ∼4 Hz and often with similarly high amplitudes within this range.

### The highest-amplitude responses under 4 Hz are not accounted for by high baseline activity/noise

It is worth addressing that it is not the case that high amplitude responses under 4 Hz is merely a consequence of higher baseline activity and noise at low frequencies. Indeed, there is a 1/*F* spectrum of baseline human brain EEG activity, such that the noise levels are particularly high at the low end of the frequency spectrum (Regan, 1989; Bach and Meigen, 1999). Specifically, the baseline amplitude is extremely high near 0 Hz and rapidly declines across very low frequencies, below ∼0.5–1 Hz (often with data processing including a high-pass frequency filter, that should be applied at about 0.01–0.1 Hz: Tanner et al., 2015); there is an ongoing modest decrease of baseline amplitude as frequency increases, with a moderate local peak in the alpha band range, ∼8–12 Hz, over the occipital cortex (Fig. 1; see also Regan, 1989; Fig. 2 of Retter and Rossion, 2016; Fig. 2 of Jacques et al., 2016; Fig. 4 of Rossion et al., 2020).

However, the noise and frequency-tagged signal do not follow the same amplitude trends across the frequency spectrum: for example, the *F* EEG response to visual stimulation over the occipital cortex peaks at ∼10 Hz, not at 3 Hz, while the noise level may be similar at these two frequencies (Fig. 1). We propose that the highest *F_s_* response amplitudes are present at low stimulation frequencies (under ∼4 Hz), but not particularly below 0.5–1 Hz, where the baseline noise level is extremely high and variable. Indeed, we have shown that the *F_s_* response amplitude is fairly stable under 4 Hz, not substantially affected by differences in baseline noise, but rather reflecting the constant amplitude of full brain responses unaffected by their successive suppression (Fig. 4; Retter and Rossion, 2016). In any event, the differences in baseline amplitude across the frequency spectrum are typically compensated for in signal measurement with a baseline correction, such as baseline subtraction or SNR. Again, it is not the case that the high-amplitude responses found to stimulation at low frequencies is merely a result of there being higher baseline activity at lower frequencies.

## Understanding the Effect of Stimulation Frequency on Response Amplitude through the Relation with Response Temporal Dynamics

Up to this point, we have targeted the misconceptions underlying the notion that visual stimulation at F = 10 Hz yields the largest-amplitude frequency-tagged brain responses. To the contrary, we have argued that it is not advantageous to target steady-state brain responses, given that acyclical responses can still be analyzed simply and advantageously in the frequency domain across multiple harmonics (specifically, through baseline-corrected amplitude summation across harmonics: *F* + 2*F* *+* 3*F…* = *F*_s_; Retter et al., 2021b). We acknowledged that stimulation at ∼10 Hz yields the highest *F* brain response but pointed out that, more importantly, it does not yield the highest *F_s_* response*.* We presented experimental evidence that when higher harmonics are appropriately taken into account in the comprehensive *F_s_* measurement, the highest-amplitude brain responses occur not to visual stimulation at ∼10 Hz, but consistently under ∼4 Hz.

In the following, we pivot to a more general discussion of the effect of stimulation frequency on brain response amplitude in relation to response temporal dynamics. The temporal dynamics of a response, including the duration of its deflections, are what define its frequency content. Temporal dynamics can be visualized directly from waveforms, and further with progressive topographies, time-frequency plots, or fitting segments of sinewaves (as in Fig. 7 of Retter et al., 2021b). In the frequency domain, temporal dynamics affect harmonic amplitude distribution (including the amplitude of *F*) and the amplitude of *F_s_*, which will be explored in the following two subsections. We continue to reason with the example of visual stimulation to human adults with high-temporal resolution recording techniques, such as EEG/MEG, over the occipital cortex.

### At low F (under ∼4 Hz), the harmonic amplitude distribution relates to response temporal dynamics

We propose that the stimulation frequency does not substantially impact the comprehensive response amplitude (*F_s_*) as long as it is relatively low (under ∼4 Hz) but that the temporal dynamics of brain responses impacts their harmonic distribution across frequencies. For example, Jacques et al. (2016) demonstrated a shorter range of harmonic responses for body parts relative to faces, mirroring the slower temporal dynamics of visual responses to body parts (see also more relative amplitude in the first harmonic for children's face individuation than faster generic face categorization: Lochy et al., 2020).

Indeed, across harmonic responses to stimulation, the amplitude at higher harmonics is not simply highest at lower stimulation frequencies but is often greater at higher individual harmonics than at *F*, particularly when low stimulation frequencies are used (McKeefry et al., 1996; Vialatte et al., 2009; Capilla et al., 2011; Tlumak et al., 2011; Winawer et al., 2013; Gaume et al., 2014). Further, the amplitude range and pattern across harmonic frequencies does not follow a universal pattern but is greatly affected by stimulus type (Jacques et al., 2016; Dzhelyova et al., 2017). In the study of Retter and Rossion (2016), it was observed that across stimulation at different F, the harmonic frequency responses were consistently characterized by the frequency at which they fell, independent of their harmonic number, and occurred within a common frequency range (see Fig. 2 of that study).

The characteristics of harmonic responses across the frequency spectrum reflect the temporal dynamics of the brain response: the peak harmonic amplitudes occur at frequencies matching the dominant frequency content of the periodic brain response, and the highest harmonic frequency occurring matches the response's highest frequency content (see Figs. 7–8 of Retter et al., 2021b; Yildirim-Keles et al., 2025). For example, with visual stimulation rates below 10 Hz, the higher harmonic amplitudes are relatively large below 10 Hz, but the amplitudes fall sharply beyond that frequency (Fig. 3*a*; van der Tweel and Lunel, 1965; Spekreijse, 1966; Regan, 1968, 1972; Başar et al., 1997; Alonso-Prieto et al., 2013). For another example, high-amplitude harmonic responses to faces occur at low frequencies with a local peak ∼6 Hz, reflecting the dominant frequency content of the face-selective response (Alonso-Prieto et al., 2013; Retter and Rossion, 2016; Rossion et al., 2020; Retter et al., 2021a). That is, stimulation at low F does not exclude recording higher harmonic responses in the “optimal” *F* range, with similar response characteristics (Herrmann, 2001; Lazarev et al., 2001).

### At higher F, the amplitude of *F* and *F_s_* relates to response temporal dynamics

As addressed previously, at high visual stimulation frequencies (approximately F > 4 Hz, sometimes extending up to F > 8 Hz), brain responses are prevented from showing complex amplitude deflections over time due to their overlapping enough to become cyclical, even approximately sinusoidal, and are represented in the frequency domain predominantly at the first harmonic, *F* (Fig. 2; Sokol, 1976 ; Regan, 1989; Alonso-Prieto et al., 2013; Zhou et al., 2016; Retter et al., 2021b). As stimulation frequency increases, the comprehensive response amplitude (*F_s_*) tends to decrease according to this successive interference (Fig. 3*b*). Once again, this understanding of the relationship of frequency-tagging response amplitude and response temporal dynamics is consistent with the interpretation of frequency-tagging generally reflecting the superpositioning of interfering (whether linear or nonlinear, constructive or deconstructive) underlying, consecutive event responses; however, it should be noted that in alternative perspectives, it has been suggested that elicited frequency-tagged oscillations may further relate to and interact with endogenous brain oscillations (discussed in Adrian and Matthews, 1934; Walter et al., 1946; Makeig et al., 2002; Heinrich, 2010; Henrich et al., 2015; Capilla et al., 2011; Gaume et al., 2019; Keitel et al., 2014).

In any event, the amplitude at *F* tends to show a complex amplitude modulation across stimulation frequencies, and its pattern is reflective of the temporal dynamics of the brain response being recorded (as recorded in the given sample, with the given methodology, at the given recording site; Heinrich, 2010; Retter et al., 2021b). In the case of visual stimulation, the highest-amplitude occipital EEG response at a single harmonic occurs at *F* = ∼10 Hz. The high response amplitude at ∼10 Hz likely relates to the underlying temporal dynamics of brain activity measured over the occipital cortex, of which prevalent, high-amplitude deflections may be represented around this frequency, and little above it, even beyond the context of frequency tagging (Fig. 2; Schürmann and Başar, 1994; Gaume et al., 2019; Chen et al., 2015; Retter et al., 2021b). As discussed in the previous section, with F below 10 Hz, the higher harmonic amplitudes have relatively large amplitudes below 10 Hz and fall sharply beyond that frequency (Fig. 3*a*).

The temporal dynamics of specific brain responses can be explored with stimulation across a range of frequencies, with the largest *F* responses indicative of the upper limits of the high-amplitude frequency content of the response and the pattern of *F_s_* response amplitude across frequencies also reflective of the frequency-content of the response (Keysers and Perrett, 2002; Alonso-Prieto et al., 2013; Yeatman and Norcia, 2016; Retter et al., 2020; 2021a; Quek et al., 2021; Marchive et al., 2024). For example, early investigations of luminance flicker discovered that the perceptual flicker fusion frequency limit was surpassed in EEG recordings at *F* and higher harmonics and may even extend up to ∼100 Hz (van der Tweel, 1964; Lyskov et al., 1998; Herrmann, 2001; Chen et al., 2015; Retter et al., 2020). For another example, *F_s_* response amplitude to stimulation of different unfamiliar faces decreases above ∼6 Hz over the right occipitotemporal cortex, while response amplitude to stimulation of a repeating facial identity may decrease above ∼2 Hz (Alonso-Prieto et al., 2013). To continue the example with face perception, responses to different faces might relate most to processing through the time window of the N170 component, i.e., from 50–200 ms after stimulus onset, such that F > approximately 6–7 Hz (1/0.15 s) might be considered “too high” (in other words, “outpacing” the brain response: Heinrich et al., 2009), while more semantic processing may occur through the time of the N400 component, up to ∼600 ms, such that the lower rate of F > approximately 2 Hz might be defined as too high (Debruille et al., 1996; Rossion and Jacques, 2011; Yildirim-Keles et al., 2025). The frequency above which response amplitude decreases relates to, and may even be predicted from, the dynamics of responses in the time domain.

## Considerations When Choosing a Stimulation Frequency

When choosing a stimulation frequency, the main two factors to consider are the following: (1) the goal of the experiment or application: e.g., are high-amplitude responses targeted, or are short assessment times key? and (2) the temporal dynamics of the targeted response, as recorded in the relevant sample, and with the applied methodology and recording site. Additional considerations include technical constraints, such as the available frequencies of the stimulation apparatus, and the comfort of participants. The following considerations are tailored to the case in which visual stimulation and high-temporal resolution recording techniques, such as EEG, MEG, electroretinogram (ERG), etc., are applied with human adults, as previously; however, the rationale of these considerations can be extended to other stimulation modalities, populations, and methodologies with different temporal dynamics.

### Stimulation at low F (under ∼4 Hz) is optimal for high-amplitude responses

Here, we propose that visual stimulation under ∼4 Hz produces the highest-amplitude human brain responses with high-temporal resolution recordings. At such low stimulation frequencies, driving multiharmonic responses, responses can be simply and advantageously measured at the baseline-corrected sum of harmonic response amplitudes, *F_s_*. Targeting high-amplitude responses in this range, which also corresponds well with the typical analysis windows of ERPs (i.e., ∼300 ms– 1s), may be advantageous. For one thing, F < 4 Hz supports that the recorded brain responses are generalizable, since they may occur fully, i.e., without suppression from interfering successive stimuli: comparable responses may be recorded within this range, e.g., with equivalent amplitude at F = 1.1 and 2.5 Hz (Fig. 4; as well as with equivalent temporal dynamics and topographies: Retter and Rossion, 2016). In this sense, the impact of stimulation frequency may be effectively removed.

Moreover, high-amplitude responses lead to high SNR and so unambiguous signal detection and improved signal classification. Likely for this reason, the term “optimal” has conventionally been used in correspondence with highest amplitude. While overall there are few studies with low F analyzing responses across harmonics (e.g., with *F*_s_), the highest-amplitude signal has been reported at low F with this approach (Retter and Rossion, 2016; Retter et al., 2020; 2021a; with auditory stimuli: Tlumak et al., 2011) and is evident in figures of experimental data (Fig. 2 of Lazarev et al., 2001; Fig. 7 of Alonso-Prieto et al., 2013; Fig. 4 of Gaume et al., 2019; Fig. 3 of Gu et al., 2024). For an example of high amplitude improving signal classification, at F = 4–5 Hz the harmonics with the highest amplitude were shown to contribute the most importantly to BCI signal classification, with a combination of harmonics yielding the best performance (Çetin et al., 2020). For another example, measuring response amplitude at *F*_s_ to words presented at F = 1 Hz enabled identification of responses in both a control and Alzheimer's disease group, even for the significantly lower responses in the Alzheimer's disease group (David et al., 2025).

Another indication that relatively low F may be considered optimal for targeting a process of interest is if responses are qualitatively modified at higher F, such as if their topography or relationship with behavioral measures are affected. For example, responses to 1 Hz oddball word stimuli were left lateralized with a base stimulation frequency from 4–10 Hz, but not at 20 Hz; a correspondence with behavioral measures of reading ability was found only at 4 Hz, but not from 6–20 Hz; these factors, in addition to the highest amplitude at 4 Hz stimulation, led the authors to conclude that this was the optimal base stimulation frequency (Marchive et al., 2024). Similarly, the characteristic (right) occipitotemporal response topography to faces is strongly evident below ∼8–12 Hz, while the response becomes less characteristic, rather more medial-occipital, above this frequency (Alonso-Prieto et al., 2013; Rossion, 2014; Rossion et al., 2020; Retter et al., 2021a).

On the lowest end, the theoretical stimulation frequency minimum is above 0 Hz for periodic stimulation. However, stimulation frequencies below ∼0.5 Hz may not be advised for practical reasons in some cases. With such low F's, longer recording times and testing sequences may be required to present a sufficient number of stimuli of interest (e.g., F = 0.3 Hz is 1 stimulus per 3.3 s) and to attempt to separate the signal well from the extremely high levels of baseline noise in the very low frequency spectrum and the noise level may be highly variable and difficult to estimate reliably. However, high amplitudes have been reported at very low stimulation frequencies with EEG (e.g., 0.67 Hz: Heinrich et al., 2009; 0.5 Hz: Vialatte et al., 2009; 0.75 Hz: Tlumak et al., 2011; 0.67 Hz: Quek and Rossion, 2017), and a practical stimulation limit may relate to the amount of noise in the signal and the recording duration.

### High F may be considered optimal in many contexts

There may be stimulation frequencies well above 4 Hz that would be advantageous in a variety of contexts, when the highest-amplitude response is not a fundamental goal, including when (1) a high information transfer rate is required; (2) sensitivity below response-amplitude ceiling is needed; and (3) differential processing of two or more frequency-tagged stimuli is targeted. In these contexts, the cost of a decrease in *F_s_* response amplitude to high F may be acceptable, as long as responses remain strong enough to be clearly disambiguated from noise in the targeted duration of recording.

In regard to a high information transfer rate, high F can be advantageous, particularly when short assessment durations (e.g., under a couple seconds) are used. For example, if aiming to rapidly monitor an attentional shift to a spatial location tagged with a flickering stimulus, a 20 Hz stimulation frequency with a high sampling rate, i.e., 20 cycles per second, may be beneficial in comparison with a slow stimulation rate like 3 Hz, i.e., three cycles per second (responses to attentional modulation have been measured successfully with a wide variety of stimulation rates: e.g., 8–12 Hz: Morgan et al., 1996; 20–28 Hz: Müller et al., 1998; 3–80 Hz: Gulbinaite et al., 2019). In regard to sensitivity below ceiling, the highest-amplitude response itself may be a negative in some cases: it limits sensitivity to individual differences that may be pronounced under challenging conditions as well as limits assessment of increased responses as a result of learning, attentional effects, or cross-modal interactions. For example, individuals’ speed of perception has also been shown not to relate to response amplitudes at low stimulation frequencies, but to a weighting of higher versus low stimulation frequency amplitudes (Retter et al., 2020: Fig. 9C-D; Retter et al., 2021a: Fig. 5).

Differential processing of two or more frequency-tagged stimuli may benefit from decreased response amplitude by successive response interference to one stimulus. For example, EEG responses to different faces cannot be distinguished from responses to repeating individual faces below 3 Hz, perhaps because slow stimulation does not encourage repetition suppression effects to the repeating identity (Alonso-Prieto et al., 2013; Rossion, 2014). Instead, despite the highest-amplitude responses occurring below 4 Hz for face stimuli, the largest amplitude difference between different facial identities versus repetitions of the same facial identity occurs at 5–6 Hz (Alonso-Prieto et al., 2013; Rossion et al., 2020; Retter et al., 2021a; with neuroimaging: Gentile and Rossion, 2014). For another example, in frequency-tagging designs of sequences of items (e.g., words in sentences: Lu et al., 2022; images of body movement: Cracco et al., 2022), responses to chunks of items, rather than individual items, may be the primary topic of investigation: if individual item responses are suppressed at high F, this might enable better dissociation of responses specific to larger sequences of items. A similar logic may be applied to the frequency-tagging oddball paradigm, in which one “oddball” stimulus type appears at a periodic rate among “base” stimuli (Heinrich et al., 2009; Norcia et al., 2015; Rossion et al., 2020): if base stimulus responses are suppressed at high F, the differential oddball response amplitude may be amplified (e.g., higher 1 Hz oddball *F_s_* responses at 4–6 vs 3 Hz base F rates: Retter et al., 2021a; effects of stimulation frequency on oddball responses are contrasted to effects of base stimulation frequency across Retter and Rossion, 2016 and Retter et al., 2020, respectively).

At the high end, F above 30–40 Hz may also be recommended in some cases. Responses to luminance may occur at higher frequencies than responses to chromaticity, enabling an isolation of luminance responses above ∼30–40 Hz (Regan, 1970; Duart et al., 2021); high-frequency luminance flicker or stimulus changes have also been introduced as a means to separate responses to such attributes from other targeted stimulus properties, such as motion or orientation, tagged at slower rates (Wattam-Bell, 1991; Braddick et al., 2005). Stimulation above 40 Hz may also be desired in the context of avoiding perceived stimulus flicker or decreasing perceptual discomfort (Lin et al., 2012; Ladouce et al., 2022; Seijdel et al., 2023; Reitelbach and Oyibo, 2024; but for alternative approaches, see, e.g., Yoshimoto et al., 2020). Very high F may also be employed to explore the limits of temporal processing dynamics (Herrmann, 2001; Keysers et al., 2001; in a “frequency-sweep” paradigm: Retter et al., 2020; 2021a; Quek et al., 2021). However, if too high F are used, it should be noted that every stimulus may not be detected (Retter et al., 2020; 2021a).

## Caveats

Up to this point, we have considered the effects of visual stimulation frequency primarily on response amplitude (overall, at *F_s_*, as well as at individual harmonics), having made several unnecessary assumptions: a single source of generic visual stimulation; a recording location over the occipital cortex; recordings with human adults; that stimulation frequency is proportional to stimulus presentation duration; and ignoring response phase. Moving beyond these assumptions introduces a number of important caveats.

We have addressed a source of generic stimulation, but the effects of F are different for repeating or changing stimuli or stimulus categories: repeating identical images may produce the highest amount of successive response interference, whereas presenting successive stimuli that are highly variable, from diverse categories, may produce the least (Keysers and Perrett, 2002; Radtke et al., 2021; Retter et al., 2020 vs Retter et al., 2021a). Parameters, such as the stimulus color, contrast range, spatial frequency, behavioral task, etc., may impact recorded responses as a function of stimulation frequency (Regan, 1966b; Sokol, 1976; Tyler et al., 1978; Regan, 1989; Fylan et al., 1997; Fawcett et al., 2004; Teng et al., 2011). In addition, there may be a substantial range of interindividual differences in temporal frequency tuning (van der Tweel and Lunel, 1965; Herbst et al., 2013; Quek et al., 2021), as well as differences across age groups (e.g., with infant brain responses weighted more toward lower frequencies than in adults, such that infants’ determined optimal F ceilings may often be much lower than adults’: Walter et al., 1946; Morrone et al., 1996; Pieh et al., 2009; Köster et al., 2023).

**Figure 5.**
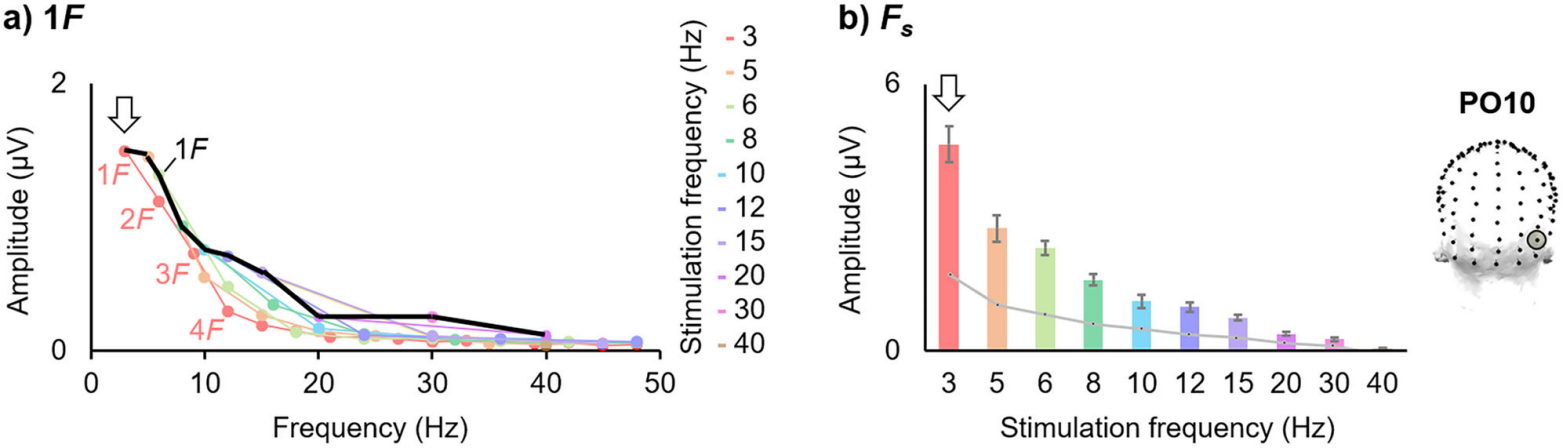
EEG responses as a function of visual stimulation frequency (PO10). Data derived from Retter et al. (2021a) (electrode PO10; *N* = 16 people; stimuli = natural face images). All notations match Figure 3. ***a***, Unlike at Oz, the amplitude of only the first harmonic (*F*, i.e., 1*F*) over the occipitotemporal cortex leads to a signal peak at 3 Hz. ***b***, The response summed across harmonics for each stimulation condition also leads to a signal peak at 3 Hz at this recording location.

Moreover, different cortical areas may differ in their frequency tuning properties, leading to different optimal stimulation rates for the activity of V1 versus V5, or V1 versus more distributed sources, for some examples (Fawcett et al., 2004; Srinivasan et al., 2006). Interestingly, the 10 Hz maximal amplitude peak at *F* of EEG recordings may be restrained to the scalp over the medial occipital cortex (around Oz), while the high comprehensive response amplitude to low stimulation frequencies, below 4 Hz, also extends over other sites, such as over the occipitotemporal cortex, at least for natural visual stimuli (Fig. 5; and manifests at the average of all high-density EEG channels: Fig. S3, Retter et al., 2020; Fig. 4, Retter et al., 2021a).

There are also some methodological considerations affecting the impact of stimulation frequency on response amplitude. For an example, F should be a submultiple of the presentation apparatus’ refresh rate and fit with full temporal integer cycles into the sequence duration (Bach and Meigen, 1999). For another example, inappropriately high-pass filtering the data (above ∼0.1 Hz: Tanner et al., 2015) can artificially decrease response amplitude at low frequencies; and inappropriately low-pass filtering the data (e.g., below 2*F*) can artificially decrease response amplitude at higher harmonic frequencies. Additionally, stimulation frequency should not be fully confounded with stimulus presentation duration, which can be adjusted in terms of the “duty cycle” of stimulus “on” and “off” time at any given frequency and may also influence response amplitude (Gauthier et al., 2012; Oralhan and Tokmakçi, 2016; Retter et al., 2018).

While we have focused on response amplitude, response phase is also an important variable to be considered. At high F, phase information at *F* reflects the latency of responses reasonably well in a single variable, although affected by successive response interference. At low F, while the interpretability of phase information across multiple harmonics is unclear at present, timing information from the phase at the first harmonic may be interpreted well (Norcia et al., 2015). Interestingly, at low F, rich temporal information can also be accessed by a return to latency information in the time domain (through a separate time-domain analysis, or an inverse-frequency transform) or in time-frequency analyses (Baker and Hess, 1984; Sieving et al., 1998; Gaume et al., 2019; Rossion et al., 2020).

## Conclusion

In concluding, we repeat that frequency-locked stimulation and analysis does not require cyclical, “steady-state” responses and a related analysis at only the first harmonic, *F*, in the frequency domain. Instead, slower stimulation rates, allowing for more complex, acyclical responses, can also be used advantageously in frequency-tagging, given that higher harmonic frequency responses are also taken into account. While the highest-amplitude response at *F* occurs at ∼10 Hz, an analysis limited to *F* misses amplitude information at higher harmonics, especially for lower stimulation frequencies, and does not correspond with time-domain amplitude ranges. The recommended combination of multiharmonic response amplitudes through baseline-corrected harmonic summation, *F*_s_, suggests that, for measuring comprehensive brain responses to visual stimuli with EEG and other high temporal-resolution recording methods, the periodic stimulation rate yielding the highest-amplitude responses is under 4 Hz, not 10 Hz. While the highest-amplitude response may not always be considered “optimal” in light of the experimental or applied goals, determining an optimal stimulation frequency requires understanding the effects of stimulation frequency on brain responses, through relation to response temporal dynamics. Ultimately, the goal of understanding the effect of stimulation frequency on brain responses is not just to determine an optimal stimulation frequency, but to enable recording brain responses that can be interpreted beyond the effect of stimulation frequency.

## Data Availability

This manuscript contains no original data.

## Synthesis

Reviewing Editor: Ifat Levy, Yale School of Medicine Decisions are customarily a result of the Reviewing Editor and the peer reviewers coming together and discussing their recommendations until a consensus is reached. When revisions are invited, a fact-based synthesis statement explaining their decision and outlining what is needed to prepare a revision will be listed below. The following reviewer(s) agreed to reveal their identity: NONE.

This opinion paper discusses the impact of frequency tagging, specifically the effect of visual stimulation frequency on brain responses. It challenges the prevailing assumption that 10-15 Hz is the optimal stimulation frequency for eliciting the highest amplitude responses, arguing instead that slower stimulation frequencies (1-3 Hz) yield more comprehensive brain responses when considering the summation of harmonic frequencies. The paper examines potential misconceptions about steady-state responses and harmonic analysis in frequency-tagging experiments, advocating for a more nuanced approach to selecting stimulation frequencies based on experimental objectives and temporal dynamics.

The topic is timely and relevant for many neuroscientists, and the authors were largely responsive to the reviews of a previous version. The paper would benefit, however, from major revisions to enhance clarity, focus, and impact, as detailed below.

- The central research question should be more explicitly stated. While it can be inferred, a clearer articulation (whether the goal is to compare single versus multiple frequencies, maximize the amplitude, or address limitations of current methods) would improve the paper's coherence.

- A stronger rationale for exploring stimulation frequency effects would enhance the paper's impact. Clarifying how maximizing the comprehensive response amplitude (Fs) improves practical applications would strengthen the argument.

- Explicitly highlighting problems that current 1F-based methods cannot resolve and demonstrating how Fs addresses these limitations would further justify the study's significance. For example, elaborating on how Fs could advance brain-computer interfaces or clinical assessments would add considerable value.

- The organization of the manuscript could be improved to enhance accessibility for a broader audience. A clearer structure might include:

1. Establishing the limitations of steady-state responses and single-frequency (1F) analysis.

2. Introducing multi-harmonic analysis (Fs) as a viable alternative.

3. Demonstrating the benefits of Fs through specific examples.

4. Concluding by linking these findings to the optimal choice of stimulation frequency.

- Please refine language for clarity and precision:

o Avoid ambiguous phrasing, such as frequency ranges "below about 4-8 Hz."

o Define all acronyms (e.g., 1F) upon first use.

o Strive for conciseness to maintain reader engagement.
